# Association of a Novel Electronic Form for Preoperative Cardiac Risk Assessment With Reduction in Cardiac Consultations and Testing: Retrospective Cohort Study

**DOI:** 10.2196/63076

**Published:** 2024-09-13

**Authors:** Mandeep Kumar, Kathryn Wilkinson, Ya-Huei Li, Rohit Masih, Mehak Gandhi, Haleh Saadat, Julie Culmone

**Affiliations:** 1 Pre-Admission Testing Center Perioperative Medicine, Hartford HealthCare Hartford, CT United States; 2 University of Connecticut Storrs, CT United States; 3 Hartford HealthCare Medical Group Hartford, CT United States; 4 Research Program Hartford HealthCare Hartford, CT United States; 5 Integrated Anesthesia Associates-Fairfield Division Hartford Healthcare Hartford, CT United States; 6 Frank H Netter MD School of Medicine Quinnipiac University North Haven, CT United States

**Keywords:** preoperative, cardiology consultations, decrease low value care, cardiology, cardiac, cohort, surgery, surgical, EMR, EMRs, EHR, EHRs, electronic medical record, electronic medical records, electronic health record, electronic health records, form, forms, assessment, assessments, risk, risks, referral, consultation, consultations, testing, diagnosis, diagnoses, diagnostic, diagnostics

## Abstract

**Background:**

Preoperative cardiac risk assessment is an integral part of preoperative evaluation; however, there is significant variation among providers, leading to inappropriate referrals for cardiology consultation or excessive low-value cardiac testing. We implemented a novel electronic medical record (EMR) form in our preoperative clinics to decrease variation.

**Objective:**

This study aimed to investigate the impact of the EMR form on the preoperative utilization of cardiology consultation and cardiac diagnostic testing (echocardiograms, stress tests, and cardiac catheterization) and evaluate postoperative outcomes.

**Methods:**

A retrospective cohort study was conducted. Patients who underwent outpatient preoperative evaluation prior to an elective surgery over 2 years were divided into 2 cohorts: from July 1, 2021, to June 30, 2022 (pre–EMR form implementation), and from July 1, 2022, to June 30, 2023 (post–EMR form implementation). Demographics, comorbidities, resource utilization, and surgical characteristics were analyzed. Propensity score matching was used to adjust for differences between the 2 cohorts. The primary outcomes were the utilization of preoperative cardiology consultation, cardiac testing, and 30-day postoperative major adverse cardiac events (MACE).

**Results:**

A total of 25,484 patients met the inclusion criteria. Propensity score matching yielded 11,645 well-matched pairs. The post–EMR form, matched cohort had lower cardiology consultation (pre–EMR form: n=2698, 23.2% vs post–EMR form: n=2088, 17.9%; *P*<.001) and echocardiogram (pre–EMR form: n=808, 6.9% vs post–EMR form: n=591, 5.1%; *P*<.001) utilization. There were no significant differences in the 30-day postoperative outcomes, including MACE (all *P*>.05). While patients with “possible indications” for cardiology consultation had higher MACE rates, the consultations did not reduce MACE risk. Most algorithm end points, except for active cardiac conditions, had MACE rates <1%.

**Conclusions:**

In this cohort study, preoperative cardiac risk assessment using a novel EMR form was associated with a significant decrease in cardiology consultation and testing utilization, with no adverse impact on postoperative outcomes. Adopting this approach may assist perioperative medicine clinicians and anesthesiologists in efficiently decreasing unnecessary preoperative resource utilization without compromising patient safety or quality of care.

## Introduction

Approximately 17.2 million surgeries are performed annually in the United States [1], with an estimated 3% combined risk of perioperative mortality, myocardial infarction (MI), and ischemic stroke [2]. Clinicians must estimate the probability of perioperative adverse events for shared decision-making and risk mitigation. This includes evaluating preexisting cardiac conditions, performing risk assessment with tools such as the Revised Cardiac Risk Index (RCRI), and using an algorithm to determine if a stress test is indicated [3]. The *American College of Cardiology /American Heart Association (ACC/AHA) Perioperative Cardiac Evaluation 2014 Guideline* [4] provides a widely accepted preoperative evaluation algorithm.

Preoperative workup may include a referral to a cardiologist, and appropriate indications for such consultations have been described [3,5]. Inappropriate cardiac testing or cardiology referrals are considered low-value care because they rarely change perioperative management, cause surgical delays, and increase costs [5-12]. Low-value preoperative cardiac stress testing is estimated to cost US $102 to US $238 million [9]. Potential causes include nonspecific referral requests or the assumption that a cardiology consultation may decrease legal risk in the event of a postoperative cardiac complication [5,13,14]. A preoperative referral to a cardiologist is an independent risk factor for low-value cardiac testing [8,14,15]. Pappas et al [16] noted significant variation in stress test orders among 118,552 patients that persisted even after adjusting for patient risk factors. Additionally, the average wait time to see a cardiologist is 26.6 days according to a 2022 AMN/Merritt Hawkins survey [17]. Studies of simulated patient scenarios have demonstrated that it is challenging for anesthesia residents [18] and practicing anesthesiologists [19] to consistently follow a preoperative cardiac algorithm. In summary, variation in requesting cardiology consultations and stress testing, unnecessary costs, and potential for surgical delays make a compelling case for an intervention to assist clinicians. However, we are not aware of any electronic medical record (EMR) process for the structured completion of a preoperative cardiac algorithm or its association with preoperative resource utilization and postoperative outcomes.

Our hospital system adapted the ACC/AHA algorithm in 2020 to standardize indications for preoperative cardiology evaluation and created an EMR form in 2022 to streamline its completion. The objective of this study was to investigate the impact of the EMR form on the preoperative utilization of cardiology consultation and cardiac diagnostic testing (echocardiograms, stress tests, and cardiac catheterization) and to evaluate postoperative outcomes.

## Methods

### Study Design, Setting, and Population

We performed a retrospective cohort analysis of patients aged ≥18 years who underwent an outpatient preoperative evaluation between July 1, 2021, and June 30, 2023, followed by an elective surgical procedure. Exclusion criteria were urgent and emergent surgical procedures, duplicate visits, and incomplete data. Hartford Healthcare is a 7-hospital integrated health care system in Connecticut. The preoperative evaluation centers are staffed by advanced practice providers, in collaboration with internal medicine hospitalist physicians. The data of interest were collected as part of routine clinical care. The study followed the STROBE (Strengthening the Reporting of Observational Studies in Epidemiology) reporting guideline [20].

### Ethical Considerations

This study was approved by the Institutional Review Board of Hartford Healthcare (HHC-2023-0113; approved on May 18, 2023), which waived the requirement for written informed consent. The data were deidentified before study analysis was performed. No compensation was provided to study participants.

### Preoperative Cardiac Risk Algorithm Used in This Study

#### Overview

**Figure 1:**
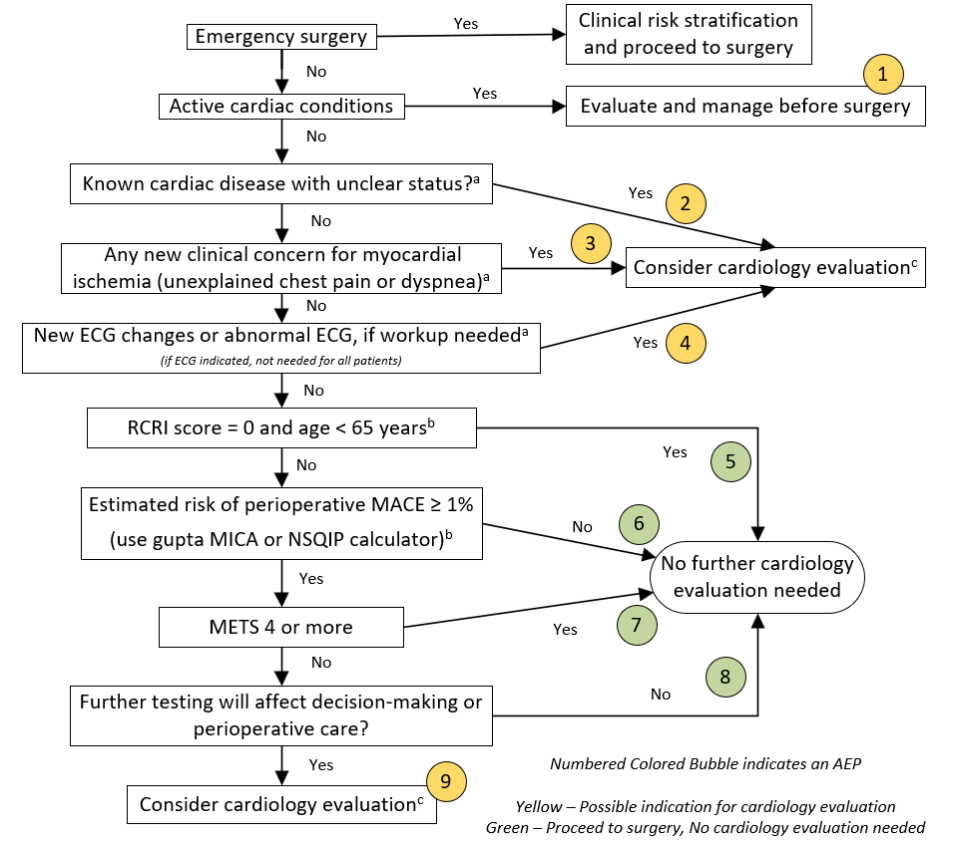
Preoperative cardiac risk assessment algorithm used in this study. a: Nonacute cardiovascular symptoms or known cardiac disease with unclear status, reasonable to consider cardiology input before surgery. b: Estimated MACE risk: those with an RCRI score of zero and age <65 years are considered low risk. The MACE risk % is calculated using the Gupta MICA or ACS NSQIP surgical risk calculator. c: Consider cardiology evaluation: our institution determined that it was optimal to defer the ordering of noninvasive stress testing to a cardiologist. ACS: American College of Surgeons; AEP: algorithm end point; ECG: electrocardiogram; MACE: major adverse cardiac events; METS: metabolic equivalents; MICA: Myocardial Infarction or Cardiac Arrest; NSQIP: National Surgical Quality Improvement Program; RCRI: Revised Cardiac Risk Index.

Our institution’s preoperative cardiac risk algorithm is adapted from the 2014 ACC/AHA perioperative cardiovascular evaluation guideline [4] with modifications to address nonacute cardiovascular symptoms, timing of intervention for coronary artery disease (CAD), stability of preexisting cardiac disease, and a nuanced consideration of major adverse cardiac events (MACE) risk, as detailed below and represented in Figure 1.

#### Nonacute Cardiovascular Symptoms or Known Cardiac Disease

The 2014 ACC/AHA algorithm does not include an assessment of nonacute cardiovascular symptoms. However, in clinical practice, potential evidence of new myocardial ischemia, such as unexplained chest pain, dyspnea, new ischemic electrocardiogram (ECG) changes, or abnormal ECG findings without prior workup, may warrant further evaluation [3,21,22]. Additionally, patients with CAD require consideration of the timing of surgery relative to the time elapsed since coronary revascularization. Finally, if the stability of preexisting cardiac disease is unclear, a cardiologist’s input can be valuable [3].

#### Estimated MACE Risk

The ACC/AHA algorithm suggests using the RCRI, Gupta Myocardial Infarction or Cardiac Arrest (MICA), or the American College of Surgeons (ACS) National Surgical Quality Improvement Program (NSQIP) surgical risk calculators. The RCRI calculator helps select low-risk patients only if RCRI score is zero and the age is <65 years, as noted in the Canadian Cardiovascular Society 2017 guideline [23], based on the Vascular Events In Noncardiac Surgery Patients Cohort Evaluation (VISION) study [24], showing increased MACE risk in patients older than 65 years, even in the absence of other risk factors. Hence, *RCRI score of 0 and age <65 years* is our algorithm’s initial step for MACE assessment [21]. The Gupta MICA and ACS NSQIP surgical risk calculators provide a more specific assessment [25] of the patient’s risk since they combine surgical and patient risk factors. Consequently, their use is in better alignment with the ACC/AHA algorithm, categorizing MACE risk <1% as low risk.

### EMR Form for Consistent Algorithm Completion

**Figure 2:**
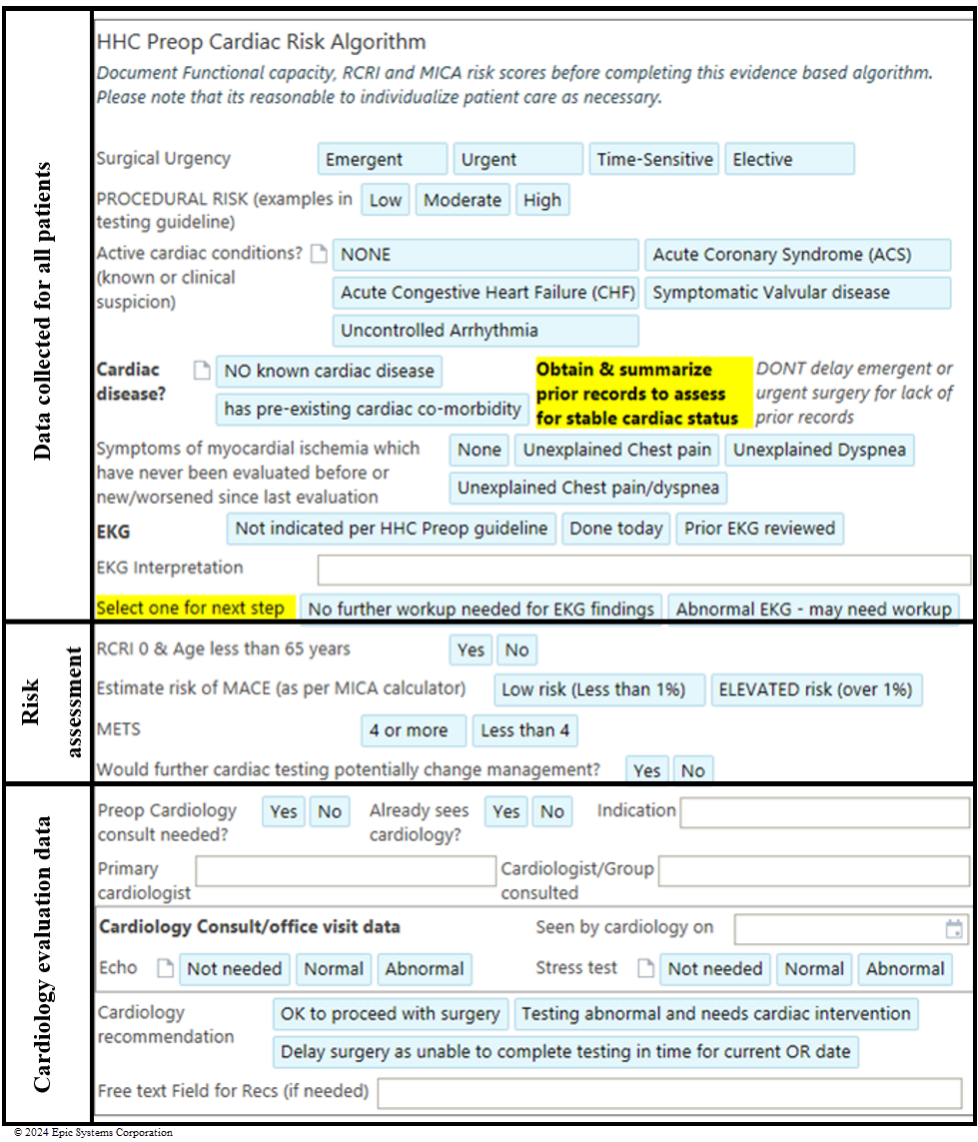
The Preop Cardiac Risk Algorithm smart form with all possible variables. HHC: Hartford Healthcare; MACE: major adverse cardiac event; MICA: Myocardial Infarction or Cardiac Arrest; RCRI: Revised Cardiac Risk Index.

In busy clinical practice, consistently completing a multistep algorithm can be challenging. To address this issue, we developed an EMR form (Epic) to assist clinicians in performing preoperative assessments (Figure 2). This takes less than 1 minute to complete; displays suggestions when preoperative cardiac testing may be unnecessary (an example is shown in Multimedia Appendix 1); and tracks the completed steps of the algorithm and the point at which it ends, referred to as the algorithm end point (AEP). The electronic form was implemented on July 1, 2022, as a standard part of outpatient preoperative evaluations performed at Hartford Healthcare preoperative evaluation centers. The form has 3 components: basic clinical information (completed for all patients), risk assessment (these steps use the preoperative risk tool results to guide the clinician through the steps of the algorithm), and cardiology consultation data (if performed). The AEPs are shown in Figure 1.

### Exposure, Variables, and Outcomes

The primary exposure was the completion of the EMR form. The 2 study cohorts were dichotomized based on the date of preoperative evaluation: from July 1, 2021, to June 30, 2022 (pre–EMR form cohort), and from July 1, 2022, to June 30, 2023 (post–EMR form cohort). The following variables were collected: demographic data (age, sex, race, ethnicity, date of the preoperative center visit, and date and type of surgery), comorbidities (atrial fibrillation, CAD, congestive heart failure, cerebrovascular accident, transient ischemic attack, chronic kidney disease, or diabetes mellitus), perioperative risk scores (functional capacity, see Multimedia Appendix 2; American Society of Anesthesiologists physical status; RCRI score; and Gupta MICA score), and surgical risk level (categorized as low, moderate, or high risk, with standard definitions used at our institution; see Multimedia Appendix 3).

The primary preoperative resource utilization outcomes were the completion of preoperative cardiology consultations and cardiac diagnostic testing (echocardiography, stress tests, or cardiac catheterization). These must have occurred within 60 days before the surgery to be considered preoperative (The 60-day timeframe was selected to account for instances where surgery is rescheduled due to delays in obtaining testing, although preoperative evaluations typically occur 30 days before surgery). The primary 30-day postoperative outcome collected were as follows: MACE (defined as a composite measure of acute MI, cardiac revascularization, acute congestive heart failure [CHF], or all-cause mortality), acute MI (as defined by the Standardized Endpoints in Perioperative Medicine initiative; see Multimedia Appendix 4) [26], cardiac revascularization (percutaneous coronary intervention or coronary artery bypass graft surgery), acute CHF (defined as clinical or radiographic evidence of volume overload treated with diuretics), and all-cause mortality. Secondary outcomes were intensive care unit (ICU) utilization, all-cause emergency department visits, and all-cause readmissions within 30 days after surgery. Mortality data were obtained from the Connecticut Department of Public Health [27]. All deaths in Connecticut are reported to the Department of Public Health; hence, we consider this a reliable measure. All other data and outcomes were extracted from EMR reporting.

The appropriateness of cardiology consultations was evaluated in the post–EMR form cohort. Possible cardiology consultation indications were defined as the presence of an active cardiac condition (AEP 1); known cardiac disease with unclear status (AEP 2); concern for myocardial ischemia (AEP 3); new ECG changes or abnormal ECG with no prior workup (AEP 4); and elevated MACE risk with poor functional capacity, for which further testing may change management (AEP 9). All other AEPs were considered “no clear indications” (Figure 1).

### Statistical Analysis

The study population was depicted with frequencies and percentages for binary or categorical information and the median and IQR for the numerical data. Group comparisons for binary or categorical information were performed using the chi-square or Fisher exact test if the sample size was small for binary variables. If *P*<.05 was observed for the first test for categorical variables (>2 classes), a post hoc test was carried out with Bonferroni adjustment. The independent-samples Mann-Whitney *U* test was used for age comparison between the groups.

Propensity score matching was performed to identify comparable subpopulations. The predictive probability of assigning patients to the pre– versus post–EMR form cohort was generated using the demographics and patient characteristics listed in the *Methods* section. Propensity score–matched, pre– and post–EMR form cohorts were identified using a 1:1 case-control match on propensity score [28], and baseline characteristics were evaluated to determine whether the 2 subpopulations were comparable. A sensitivity analysis was conducted using the E-value approach to assess the magnitude of the unmeasured confounding bias [29,30]. The lowest E-value is 1, suggesting that no unmeasured confounding exists to explain the current association between the predictor and outcome. A higher E-value indicates a stronger unmeasured confounder association that may explain the current effect [29]. A subanalysis was performed in the post–EMR form cohort to evaluate the association of appropriate cardiology consultation indications versus not with completed cardiology consultations and 30-day MACE. Hypothesis testing was performed with a 2-sided α of .05, and all analyses were performed using IBM SPSS Statistics for Windows (version 29).

## Results

Between July 1, 2021, and June 30, 2023, a total of 26,583 sequential outpatient preoperative evaluations met the inclusion criteria. Duplicate visits (n=442) and patients with missing preoperative risk scores (n=657) were excluded. The final study population comprised 25,484 patients: 13,365 before and 12,119 after the EMR form implementation. Unadjusted analysis showed that the 2 cohorts were significantly different in terms of several baseline characteristics. A higher proportion of patients in the preintervention group were male (5987/13,365, 44.8% vs 5279/12,119, 43.6%; *P*=.047), were White (10,613/13,365, 79.4% vs 9407/12,119, 77.6%; *P*=.02), had a higher baseline incidence of atrial fibrillation (1170/13,365, 8.8% vs 976/12,119, 8.1%; *P*=.04), and had CAD (1516/13,365, 11.3% vs 1246/12,119, 10.3%; *P*=.006) compared to the postintervention group. Additionally, the pre–EMR form group had a higher number of patients with poor functional capacity (2187/13,365, 16.4% vs 1770/12,119, 14.6%; *P*<.001) and patients who were undergoing high-risk surgery (3071/13,365, 23% vs 2527/12,119, 20.9%; *P*<.001). Propensity score matching resulted in 11,645 matched pairs (23,290/25,484, 91.4% of the full cohort) with similar pre– and post–EMR form cohorts in terms of demographics, comorbidities, perioperative risk tool results, and surgical risk levels, as there were no statistically significant differences (all *P*>.05; Table 1).

**Table 1:** Characteristics of unmatched and propensity score–matched cohorts by electronic medical record (EMR) form implementation status.

Variables			Unmatched cohort			*P* value^a^		Propensity score cohort					*P* value^a^
			Pre–EMR form (n=13,365)	Post–EMR form (n=12,119)			Pre–EMR form (n=11,645)		Post–EMR form (n=11,645)				
**Demographics**													
	**Age (years), median (IQR)**		64 (53-72)	64 (53-72)	.39		64 (53-72)		64 (53-72)		.72		
	**Sex, n (%)**					*.047^b^*						.62	
		Male	5987 (44.8)	5279 (43.6)			5115 (43.9)		5077 (43.6)				
		Female	7377 (55.2)	6839 (56.4)			6530 (56.1)		6568 (56.4)				
	**Race, n (%)**					*.02*						.96	
		Asian	143 (1.1)	149 (1.2)			134 (1.2)		145 (1.2)				
		African American	989 (7.4)	976 (8.1)			911 (7.8)		925 (7.9)				
		White	10,613 (79.4)	9407 (77.6)			9245 (79.4)		9223 (79.2)				
		American Indian	42 (0.3)	43 (0.4)			42 (0.4)		41 (0.4)				
		Others	1578 (11.8)	1544 (12.7)			1313 (11.3)		1311 (11.3)				
	**Hispanic or Latino, n (%)**		1540 (11.8)	1418 (12)	.50		1374 (11.8)		1391 (11.9)		.73		
	**Comorbidities, n (%)**												
		Atrial fibrillation	1170 (8.8)	976 (8.1)	*.04*		930 (8)		949 (8.1)		.65		
		Coronary artery disease	1516 (11.3)	1246 (10.3)	*.006*		1203 (10.3)		1210 (10.4)		.88		
		Congestive heart failure	465 (3.5)	407 (3.4)	.60		384 (3.3)		388 (3.3)		.88		
		CVA^c^ or TIA^d^ history	576 (4.3)	489 (4.0)	.27		452 (3.9)		465 (4)		.66		
		Chronic kidney disease	1499 (11.2)	1215 (10)	*.002*		1173 (10.1)		1187 (10.2)		.76		
		Diabetes mellitus	2735 (20.5)	2445 (20.2)	.57		2334 (20)		2357 (20.2)		.71		
		Any cardiac comorbidites^e^	5133 (38.4)	4442 (36.7)	*.004*		4266 (36.6)		4298 (36.9)		.66		
**Perioperative risk tool results, n (%)**													
	**Metabolic equivalents**					*<.001*						.49	
		Less than 4	2187 (16.4)	1770 (14.6)			1687 (14.5)		1724 (14.8)				
		4 or more	11,178 (83.6)	10349 (85.4)			9958 (85.5)		9921 (85.2)				
	**ASA^f^ physical status classification**					.79						.27	
		ASA 1 or 2	8675 (64.9)	7847 (64.7)			7618 (65.4)		7538 (64.7)				
		ASA 3 or 4	4690 (35.1)	4272 (35.3)			4027 (34.6)		4107 (35.3)				
	**Revised Cardiac Risk Index**					.38						.71	
		0 or 1	12,642 (94.6)	11,433 (94.3)			11,010 (94.5)		10,997 (94.4)				
		2 or more	723 (5.4)	686 (5.7)			635 (5.5)		648 (5.6)				
	**Gupta MICA^g^**					.93						.76	
		Low risk (less than 1%)	12,700 (95)	11,519 (95)			11,062 (95)		11,072 (95.1)				
		Elevated risk (over 1%)	665 (5)	600 (5)			583 (5)		573 (4.9)				
	**Surgical risk level**					*<.001*						.89	
		Low	2995 (22.4)	2838 (23.4)			2693 (23.1)		2720 (23.4)				
		Moderate	7299 (54.6)	6754 (55.7)			6519 (56)		6486 (55.7)				
		High	3071 (23)	2527 (20.9)			2433 (20.9)		2439 (20.9)				

^a^*P* value compares pre– vs post–EMR form implementation.

^b^Italics indicates a statistically significant difference (*P*<.05).

^c^CVA: cerebrovascular accident.

^d^TIA: transient ischemic attack.

^e^Any cardiac risk comorbidities is a composite measure of the presence of either of the following: atrial fibrillation, coronary artery disease, congestive heart failure, CVA or TIA, chronic kidney disease, or diabetes mellitus.

^f^ASA: American Society of Anesthesiologists.

^g^MICA: Myocardial Infarction and Cardiac Arrest.

**Figure 3:**
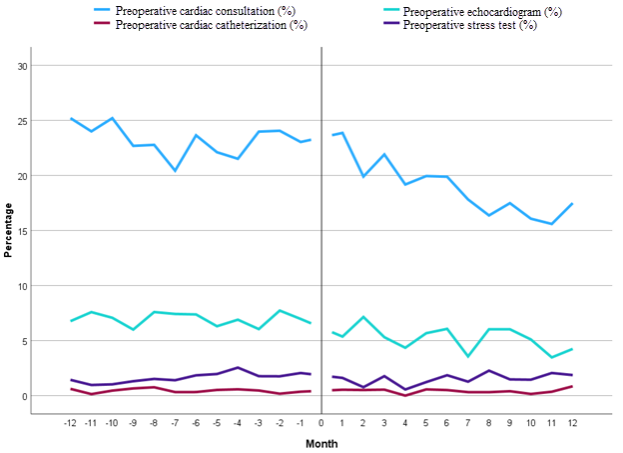
Preoperative cardiology consultations and testing percentages over the 2-year study period. "0" represents July 1, 2022 (the date of the implementation of the electronic medical record form).

In the matched cohort, cardiology consultation utilization was lower in the post–EMR form cohort (pre–EMR form: 2698/11,645, 23.2% vs post–EMR form: 2088/11,645, 17.9%; *P*<.001). Echocardiograms were completed less often in the post–EMR form cohort (pre–EMR form: 808/11,645, 6.9% vs post–EMR form: 591/11,645, 5.1%; *P*<.001). The rates of stress tests and cardiac catheterization were lower in the post–EMR form cohort; however, these differences were not statistically significant (*P*=.38 and .41, respectively). The E-values for preoperative cardiology consultation and testing ranged from 1.42 to 2.14, suggesting a low likelihood of unmeasured confounding (Table 2). Monthly trends in preoperative resource utilization are presented in Figure 3.

The 30-day postoperative outcomes were compared between the matched cohorts. No statistically significant differences were observed in the occurrence of acute MI, cardiac revascularization, acute CHF, ICU utilization, emergency department visits, readmission, or mortality (all *P*>.05; Table 3).

Preoperative cardiology consultation indications were dichotomized into “possible indications” and “no clear indications.” A higher number of patients in “possible indication” group experienced MACE as compared to those in “no clear indication” group (28/3749, 0.7% vs 18/7896, 0.2%; *P*<.001). However, the completion of preoperative cardiology consultation was not associated with a decrease in MACE risk in either group (Table 4).

The MACE count was analyzed for each AEP in the post–EMR form cohort. Active cardiac conditions were associated with 3.9% (2/51) MACE. All other AEPs had either zero or <1% MACE. Of note, *RCRI score=0 and age <65 years* was associated with 0.1% (2/3826) MACE, and MICA low risk was associated with 0.5% (16/3111) MACE. Statistical significance was noted (*P*<.001) but with low confidence, as the MACE rate was zero for several AEPs (Multimedia Appendix 5).

**Table 2:** Preoperative cardiology consultation and testing within 60 days before surgery in propensity score–matched, pre– and post–electronic medical record (EMR) form implementation cohorts.

Variables	Total (n=23,290)	EMR form implementation			*P* value^a^		E-value
		Pre–EMR form (n=11,645)	Post–EMR form (n=11,645)				
Preoperative cardiac consultation, n (%)	4786 (20.5)	2698 (23.2)	2088 (17.9)	*<.001^b^*		1.63	
Preoperative echocardiogram, n (%)	1399 (6)	808 (6.9)	591 (5.1)	*<.001*		2.14	
Preoperative stress test, n (%)	379 (1.6)	198 (1.7)	181 (1.6)	.38		1.42	
Preoperative cardiac catheterization, n (%)	96 (0.4)	52 (0.4)	44 (0.4)	.41		1.65	

^a^*P* value compares pre– vs post–EMR form implementation using the Pearson chi-square test.

^b^Italics indicates a statistically significant difference (*P*<.05).

**Table 3:** 30-Day postoperative outcomes in propensity score–matched, pre– vs post–electronic medical record (EMR) form implementation cohorts.

Variables	Total (n=23,290)	EMR form implementation			*P* value^a^
		Pre–EMR form (n=11,645)	Post–EMR form (n=11,645)		
Acute MI^b^, n (%)	28 (0.1)	14 (0.1)	14 (0.1)	>.99	
Cardiac revascularization, n (%)	7 (0)	2 (0)	5 (0)	.45	
Acute CHF^c^, n (%)	46 (0.2)	22 (0.2)	24 (0.2)	.77	
Mortality, n (%)	32 (0.1)	16 (0.1)	16 (0.1)	>.99	
MACE^d,e^, n (%)	91 (0.4)	45 (0.4)	46 (0.4)	.92	
ICU^f^ utilization, n (%)	352 (1.5)	169 (1.5)	183 (1.6)	.45	
Emergency department visit, n (%)	1308 (5.6)	676 (5.8)	632 (5.4)	.21	
Readmission, n (%)	1505 (6.5)	747 (6.4)	758 (6.5)	.77	

^a^*P* value compares pre– vs post–EMR form implementation using the Pearson chi-square test.

^b^MI: myocardial infarction.

^c^CHF: congestive heart failure.

^d^MACE: major adverse cardiac events.

^e^30-Day MACE is a composite measure of acute MI, cardiac revascularization, acute CHF, or all-cause mortality occurring within 30 days of the index procedure. Some patients had more than 1 event; hence, the composite total does not equal a simple addition of the 4 individual components.

^f^ICU: intensive care unit.

**Table 4:** 30-Day major adverse cardiac events (MACE) in the post–electronic medical record (EMR) form cohort, stratified by consultation indication and preoperative cardiology consultations.

Algorithm end point composite			Preoperative cardiac consultation, n (%)				*P* value^a^
		No (n=9557)		Yes (n=2088)			
**No clear consultation indications (n=7896)**			7180 (90.9)		716 (9.1)		—^b^
	MACE	14 (0.2)		4 (0.6)		.052	
**Possible indication for consultation (n=3749)**			2377 (63.4)		1372 (36.6)		—
	MACE	16 (0.7)		12 (0.9)		.49	

^a^*P* value compares with vs without preoperative cardiac consultation using the Pearson chi-square test.

^b^Not applicable.

## Discussion

### Principal Findings

In this cohort study of patients presenting for outpatient preoperative evaluations before surgery, completion of a structured, EMR-based preoperative cardiac algorithm was associated with a decreased frequency of preoperative cardiology consultations and echocardiograms without an increase in postoperative MACE and other adverse outcomes.

Our study was observational; however, several factors support the validity of our results. We studied a considerable surgical population over 2 years and used propensity score matching to balance several potential confounders of perioperative risk between cohorts, including age, sex, race, comorbidities, perioperative risk tool results, and inherent surgery-specific risks. Both cohorts had a substantial burden of comorbidities (~36%), and a high proportion of patients underwent moderate- or high-risk surgical procedures (~76%). The postoperative outcomes were similar between the pre– and post–EMR form cohorts, with a cumulative low risk of postoperative MACE of 0.4% (pre–EMR form: 45/11,645, 0.4% vs post–EMR form: 46/11,645, 0.4%; *P*=.92), suggesting that our initiative decreased unnecessary consultations and testing while maintaining an excellent quality of care. Consistent with our results, several other studies also show that inappropriate cardiology consultations and stress tests do not lower the risk of postoperative MACE [5-8,10-12].

A subanalysis of “appropriate” versus “no clear indications” for cardiology consultation in the postintervention cohort showed that many consultations were still requested without a clear indication, highlighting an opportunity to improve the process. Interestingly, the MACE rates did not differ regardless of whether a cardiology consultation was completed, even when there was an appropriate reason for the consultation (Table 4). Similar findings have been reported in the context of preoperative cardiology consultations in patients hospitalized for hip fracture surgery [8]. We suggest that preoperative cardiology consultations should be requested only if required for clinical management and not just because a surgical procedure is planned.

Our study provides a template to guide clinicians in adhering to preoperative algorithms to reduce low-value care. Since the EMR form data can be used to determine the algorithm steps completed, future research could use a similar process to evaluate the ACC/AHA algorithm [4], which has not been prospectively validated despite its wide use.

Our study had several limitations. Due to the retrospective design, the possibility of selection bias and residual confounding remains despite balancing the measured baseline characteristics using propensity scoring. However, we also calculated the E-value, which suggests a low likelihood of unmeasured confounders. The high baseline rate of preoperative cardiology consultations in our study population (23%) may not reflect clinical practice elsewhere. However, our literature review shows a significant variation with rates of 8.7% in low-risk gastrointestinal endoscopic procedures [7], 51.8% in a population undergoing low-risk bariatric surgery [15], and from 6.9% to 87.5% in a study of patients undergoing vascular surgery across 29 hospitals [31]. Our study observed a lower rate of complications compared to NSQIP data [32]; however, NSQIP uses random sampling [33] as compared to all consecutive patients in our study, including approximately 25% of patients undergoing low-risk surgical procedures. Lastly, our data are from a single health care system and thus may not be generalizable to other care settings.

### Conclusion

The use of a novel electronic form for the preoperative cardiac risk algorithm is associated with decreased cardiology consultations and testing without an increase in postoperative cardiac complications.

## Appendix

Multimedia Appendix 1 Example of algorithm ending when Myocardial Infarction or Cardiac Arrest (MICA) risk is <1%.

Multimedia Appendix 2 Functional capacity assessment.

Multimedia Appendix 3 Risk classification of surgical procedures.

Multimedia Appendix 4 Acute myocardial infarction determination methodology.

Multimedia Appendix 5 30-Day major adverse cardiac events (MACE) for each algorithm end point (AEP), post-EMR cohort only. EMR: electronic medical record.

## Abbreviations

ACC: American College of Cardiology
ACS: American College of Surgeons
AEP: algorithm end point
AHA: American Heart Association
CAD: coronary artery disease
CHF: congestive heart failure
ECG: electrocardiogram
EMR: electronic medical record
ICU: intensive care unit
MACE: major adverse cardiac events
MI: myocardial infarction
MICA: Myocardial Infarction or Cardiac Arrest
NSQIP: National Surgical Quality Improvement Program
RCRI: Revised Cardiac Risk Index
STROBE: Strengthening the Reporting of Observational Studies in Epidemiology
VISION: Vascular Events In Noncardiac Surgery Patients Cohort Evaluation
