# Supplementary material for: Association of a Novel Electronic Form for Preoperative Cardiac Risk Assessment With Reduction in Cardiac Consultations and Testing: Retrospective Cohort Study
Source: JMIR Perioper Med. 2024 Sep 13;7:e63076. doi: 10.2196/63076 (PMC11437228; doi:10.2196/63076)
Supplement: Multimedia Appendix 1 [file periop_v7i1e63076_app1.pdf]

Example of Algorithm ending when MICA risk is less than 1%

The dark-shaded buttons indicate the selected options for each variable. Once the appropriate endpoint is reached, the form does not show further options because they are not applicable; in this example, the METS and subsequent questions are not displayed to the user.

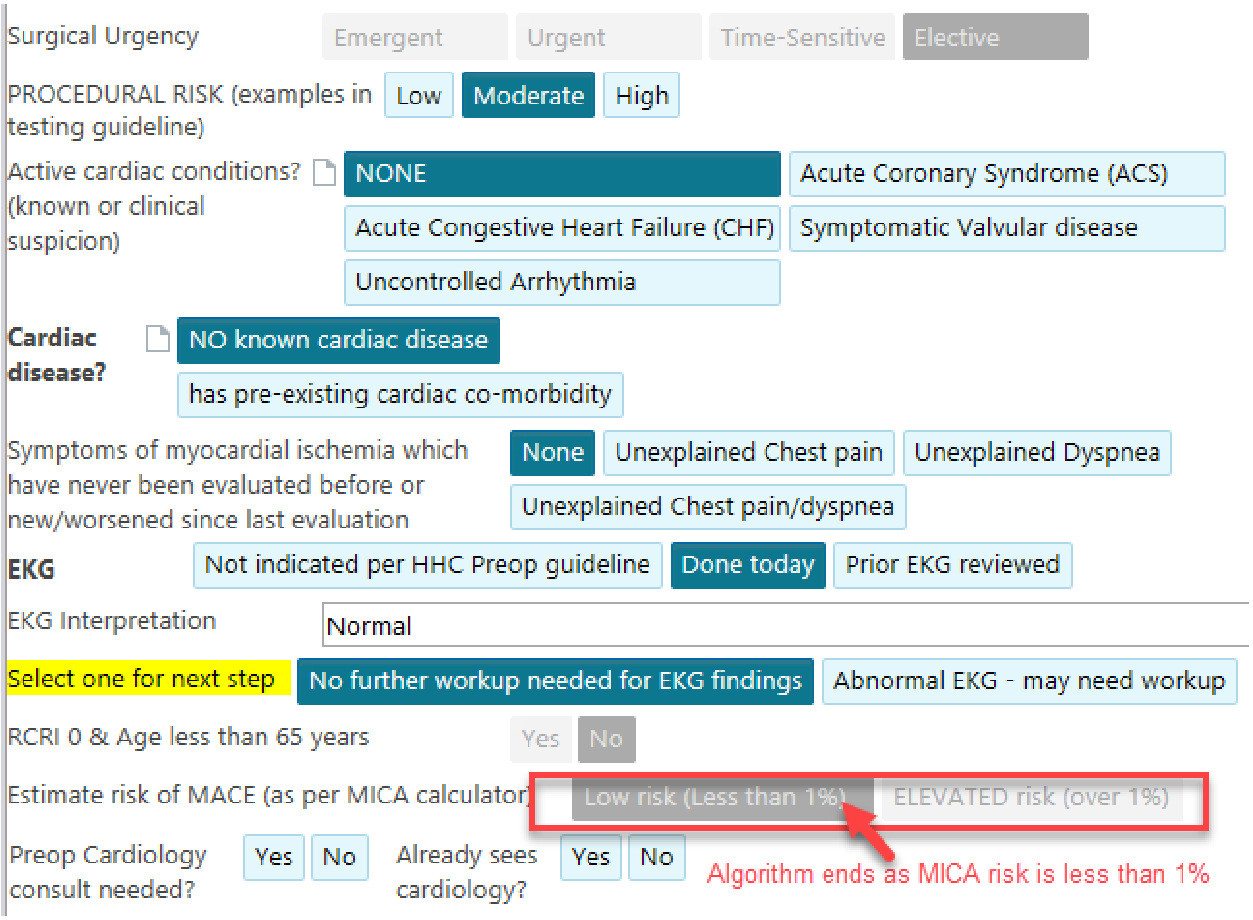

<u>Sample text that appears in the note to guide the provider</u>

PREOP CARDIAC RISK ASSESSMENT
Planned for Elective, Moderate risk surgical procedure. No active cardiac conditions. NO known cardiac disease. No chest pain or dyspnea at rest or exertion.

EKG: <u>Done today</u>. Tracing reviewed by me & my interpretation is <u>Normal</u>.

Low risk (Less than 1%) of MACE as per Gupta MICA cardiac risk calculator, No further preoperative cardiac testing is indicated.

© 2024 Epic Systems Corporation
