# Supplementary material for: Association of a Novel Electronic Form for Preoperative Cardiac Risk Assessment With Reduction in Cardiac Consultations and Testing: Retrospective Cohort Study
Source: JMIR Perioper Med. 2024 Sep 13;7:e63076. doi: 10.2196/63076 (PMC11437228; doi:10.2196/63076)
Supplement: Multimedia Appendix 2 [file periop_v7i1e63076_app2.pdf]

Functional capacity assessment

Functional capacity was assessed by documenting the highest level of self-reported activity achieved by the patient at baseline. The corresponding levels of approximate metabolic equivalents (METs) are shown in parentheses.

Functional capacity activity descriptions

1. Bedbound or dependent with ADLs (1 MET)
2. Can take care of self, such as eat, dress, etc. (1 MET)
3. Can walk up a flight of steps or a hill (4 METs)
4. Can do heavy work or climb two flights of stairs (between 4 and 10 METs)
5. Can participate in strenuous sports (>10 METs)

Functional capacity was dichotomized as “less than 4” and “4 or more” using the cutoff of “under 4 METs” as poor functional capacity
