# Supplementary material for: Association of a Novel Electronic Form for Preoperative Cardiac Risk Assessment With Reduction in Cardiac Consultations and Testing: Retrospective Cohort Study
Source: JMIR Perioper Med. 2024 Sep 13;7:e63076. doi: 10.2196/63076 (PMC11437228; doi:10.2196/63076)
Supplement: Multimedia Appendix 3 [file periop_v7i1e63076_app3.pdf]

Risk classification of surgical procedures (examples, not an exhaustive list)

<table>
<tr><th colspan="2">LOW RISK</th></tr>
<tr><th>Category 1- Minimally Invasive</th><th>Category 2- Minimal to Moderately Invasive
~Blood loss less than 500 mL</th></tr>
<tr><td>Breast Biopsy / Lumpectomy
Minor skin or subcutaneous lesion
Myringotomy tubes
Cystoscopy (except in the setting of sepsis)
Vasectomy
Circumcision
Fiberoptic Bronchoscopy
Cataract</td><td>Laparoscopic Cholecystectomy
Diagnostic Laparoscopy, Lysis of Adhesions
D&C (dilation and curettage), Tubal Ligation
Arthroscopy
Inguinal Hernia / Umbilical Hernia Repair
Tonsillectomy /Adenoidectomy
Septoplasty / Rhinoplasty
Percutaneous Lung Biopsy</td></tr>
<tr><th colspan="2">MODERATE RISK</th></tr>
<tr><th colspan="2">Category 3- Moderately to Significantly Invasive
~Blood loss 500-1500mL</th></tr>
<tr><td colspan="2">• Mastectomy / Breast Reconstruction
• Thyroidectomy / Parathyroidectomy
• Cesarean section
• Hysterectomy, Myomectomy (benign disease)
• Open cholecystectomy
• Primary Hip/Knee joint replacement
• Major Laparoscopic procedures
• Bariatric /Weight Reduction Surgery
• Resection/Reconstruction of gastrointestinal (GI) tract</td></tr>
<tr><th colspan="2">HIGH RISK</th></tr>
<tr><th>Category 4- Highly Invasive
~Blood loss GREATER than 1500mL</th><th>Category 5- Highly Invasive
~Blood loss GREATER than 1500mL</th></tr>
<tr><td>• Spine surgeries
• Major orthopedic (revision joint replacement, hip/pelvic fracture, oncology)
• Major reconstruction of GI tract
• Major Urologic (prostatectomy, nephrectomy, cystectomy, ileal conduit)
• Major Oncologic (Surg Oncology, Gynaecology oncology, Thoracic)
• Major Vascular reconstruction</td><td>• Post-op ICU planned

• Cardiothoracic procedure
• Intracranial procedure
• Major ENT (ear nose throat) procedure
• Major vascular, skeletal, neurologic repair</td></tr>
</table>
