# Supplementary material for: Association of a Novel Electronic Form for Preoperative Cardiac Risk Assessment With Reduction in Cardiac Consultations and Testing: Retrospective Cohort Study
Source: JMIR Perioper Med. 2024 Sep 13;7:e63076. doi: 10.2196/63076 (PMC11437228; doi:10.2196/63076)
Supplement: Multimedia Appendix 4 [file periop_v7i1e63076_app4.pdf]

Acute Myocardial infarction (Acute MI) determination methodology

We used the StEP initiative definition for Acute MI.

Definition: Acute myocardial injury with clinical evidence of acute myocardial ischaemia and with detection of an increase or decrease in cardiac troponin values with at least one value above the 99th percentile URL and at least one of the following:

1. Symptoms of myocardial ischemia
2. New ischaemic ECG changes
3. Development of pathological Q waves
4. Imaging evidence of new loss of viable myocardium or new regional wall motion abnormality in a pattern consistent with an ischaemic etiology
5. Identification of a coronary thrombus by angiography or autopsy

Methodology: Electronic medical records of study patients were queried for billing diagnosis codes of Acute Myocardial Infarction and abnormal troponin values documented from the day of surgery to 30 days afterwards. Any charts with the presence of either were reviewed by the study personnel, and the StEP definition was used to confirm the diagnosis.

Billing diagnosis group codes for Acute MI	
ICD-9	410.00 to .XX
ICD-10	I21.00 to .XX, I22.00 to .XX & I24.00 to .XX

Assay utilized at all Hartford Healthcare hospitals – High sensitivity troponin T by Roche diagnostics, 99th percentile upper reference limit is 22 ng/L.

Reference

Beattie WS, Lalu M, Bocock M, et al. Systematic review and consensus definitions for the Standardized Endpoints in Perioperative Medicine (StEP) initiative: cardiovascular outcomes. Br J Anaesth. 2021;126(1):56-66. doi:10.1016/j.bja.2020.09.023
