# Supplementary material for: Association of a Novel Electronic Form for Preoperative Cardiac Risk Assessment With Reduction in Cardiac Consultations and Testing: Retrospective Cohort Study
Source: JMIR Perioper Med. 2024 Sep 13;7:e63076. doi: 10.2196/63076 (PMC11437228; doi:10.2196/63076)
Supplement: Multimedia Appendix 5 [file periop_v7i1e63076_app5.pdf]

30-day Major adverse cardiac events (MACE) for each Algorithm End Point (AEP) – Post EMR cohort only

		MACE[a] count (%)		
AEP #	Algorithm End Point (AEP) description	No	Yes	Total
1	Active cardiac condition Present*	49 (96.1%)	2 (3.9%)	51
2	Known cardiac disease with unclear status*	3115 (99.3%)	21 (0.7%)	3136
3	Possible new Myocardial Ischemia symptoms*	82 (100%)	0 (0%)	82
4	Abnormal ECG - may need workup*	474 (99.9%)	5 (0.1%)	479
5	RCRI 0 and Age less than 65	3824 (99.9%)	2 (0.1%)	3826
6	MICA - Low risk (less than 1%)	3095 (99.5%)	16 (0.5%)	3111
7	MICA elevated (over 1%), but METS ≥ 4	82 (100%)	0 (0%)	82
8	METS < 4, but further testing will not change management	21 (100%)	0 (0%)	21
9	METS < 4 and further testing may change management*	1 (100%)	0 (0%)	1
10	Provider discretion[b] – Consult requested	126 (100%)	0 (0%)	126
11	Provider discretion[b] – No consult	730 (100%)	0 (0%)	730
	Total	11599 (99.6%)	46 (0.4%)	11645

Note

a: 30-day MACE is a composite measure of Acute MI, Cardiac revascularization, Acute CHF, or All-cause mortality occurring within 30 days of the index procedure

b: Patients with Algorithm data completed but not falling in a particular AEP were grouped as "Provider discretion" as there wasn't a clear reason for providers to have requested a cardiology consultation

***:** These are considered possible indications for a cardiology consultation. All other AEPs are considered "no clear consult indication."

The differences in MACE for each step are statistically significant (P-value <0.001) but with low confidence as 8 cells (40%) had expected count less than 5.

Abbreviations
MACE; Major adverse cardiac event, AEP; Algorithm End Point, EMR; Electronic Medical Record, ECG; Electrocardiogram, RCRI; Revised Cardiac Risk Index, MICA; Gupta Myocardial infarction and cardiac arrest score, METS; Metabolic Equivalents, MI; Myocardial infarction, CHF; Congestive Heart Failure
